# Echocardiographic assessment of right ventricular function: current clinical practice

**DOI:** 10.1007/s10554-018-1428-8

**Published:** 2018-09-06

**Authors:** Matthias Schneider, Stefan Aschauer, Julia Mascherbauer, Hong Ran, Christina Binder, Irene Lang, Georg Goliasch, Thomas Binder

**Affiliations:** 1 Department of Internal Medicine II, Medical University of Vienna, Waehringer Guertel 18-20, 1090 Vienna, Austria; 2 Department of Echocardiography, Nanjing First Hospital Affiliated to Nanjing Medical University, Nanjing, China

**Keywords:** Right ventricular function, Transthoracic echocardiography, TAPSE, Eyeballing

## Abstract

Echocardiographic evaluation of right ventricular (RV) function is a challenge due to the complex anatomy of the RV. Several transthoracic echocardiographic methods have been suggested for the quantification of RV function. However, many of the parameters are time consuming and need dedicated hardware and software. We suspected that the majority of the established markers are not used on a wide basis. In a multinational online survey, we evaluated the use of current clinical standards for the quantification of RV function in clinical practice. Through the network of an Ultrasound Online Teaching Platform, echocardiographers were invited to participate in an open online survey. The participants were asked about the parameters (eyeballing, TAPSE, S′, fractional area change, RIMP, 3D-EF, dp/dt, longitudinal strain) they used in clinical practice. A total of 1150 participants from 109 countries completed the survey. Only eyeballing (72%), TAPSE (69%), and S′ (31%) were commonly used in clinical routine. These methods were applied significantly less common in low-income economies when compared to high-income economies. Twenty-three percent of all participants stated to rely on eyeballing only, when evaluating RV function in clinical routine. New technologies, such as global longitudinal strain (3%) and 3D echocardiography (1%) were rarely applied independent of region and economic strength. Eyeballing and TAPSE are the most widely used methods in echocardiography for the assessment of RV function. Although advanced parameters such as longitudinal strain and 3D echocardiography were shown to be highly accurate, they are rarely used in clinical routine.

## Introduction

Correct assessment of right ventricular (RV) function is an essential part of every transthoracic echocardiographic (TTE) examination. It plays an important role in the diagnosis and management of many diseases and conditions.

TTE evaluation of RV function (RVF) remains challenging due to the complex anatomy of the RV. In contrast to the left ventricle, there is no exact geometric model for the RV that would permit volumetric measurements based on 2D echocardiographic images. Right ventricular ejection fraction (RVEF) derived from cardiac magnetic resonance imaging (MRI) remains the gold standard for functional RV assessment.

Several parameters have been suggested for echocardiographic evaluation of the RV. The simplest and most commonly used parameter is visual assessment. The reliability of visual quantification has been evaluated in previous studies [1, 2]; the authors concluded that eyeballing alone is insufficient to quantify RV function. However, factors influencing visual assessment and its actual use in various parts of the world remain ambiguous.

Guidelines for TTE assessment of the right heart demand the use of at least one quantitative parameter in addition to visual gradation [3]. Several studies have demonstrated the value of tricuspid annular plane systolic excursion (TAPSE) [4–6], tissue Doppler imaging of the basal free lateral wall of the RV (S′) [7], longitudinal strain of the free lateral wall of the right ventricle (RV-GLS) [8–10], fractional area change (FAC) [11–14], right ventricular myocardial performance index (RIMP) [7, 15], and the rate of pressure rise in the RV (dp/dt) [16]. Furthermore, three-dimensional echocardiography allows an exact and reproducible estimation of volume and ejection fraction of the RV [17–20].

However, we lack knowledge about the gap between guideline recommendations and the actual assessment of RV function in clinical practice. For the present study, we performed a multinational online survey to determine those methods most frequently used by echocardiographers for the quantification of RV function.

## Materials and methods

Through the network of an Ultrasound Teaching Platform (123sonography.com), echocardiographers were invited to participate in an open online survey. All answers received between April 1 and July 31 2017 were analyzed. The study was approved by the ethics committee of the Medical University of Vienna (EK #1288/2016). The study protocol is in conformity with the ethical guidelines of the Declaration of Helsinki.

### Questionnaire design

The participant’s name and the name of the institution were not included in the questionnaire. Baseline demographic data were obtained, such as age, the country in which participants practiced, profession, work setting (university hospital, hospital, private clinic/private office, other), and experience in TTE.

Participants were asked about the parameters they used for the assessment of right ventricular function. They answered two questions, (1) which methods of RV quantification they apply occasionally as part of their individual diagnostic repertoire, and (2) which methods they apply in daily clinical routine on most of their examinations.

Common methods (eyeballing, TAPSE, S′, FAC, RIMP, 3D-EF, dp/dt, and RV-GLS) were given as possible answers in a multiple-choice format. When applicable, the respondents could mention other techniques in a free-response box.

### Objectives

The main objective of this study was to determine which methods for the evaluation of right ventricular function are applied in daily clinical practice in a real-life sample of echocardiographers from all over the world.

### Different regions of the world and differences between low and high income economies

The World Bank divides the countries of the world into four income classes depending on the gross national income per capita ranking (GNI). Low income economies were defined as those with a GNI of $1005 or less in 2016. Lower middle-income economies were defined as those with a GNI between $1006 and $3955. Upper middle-income economies were defined as those with a GNI between $3956 and $12,235. High-income economies were defined as those with a GNI of above $12,235 [21].

According to the World Bank, we divided the world into the following seven regions to analyze regional differences: East Asia and Pacific, Europe and Central Asia, Latin America and the Caribbean, Middle East and North Africa, North America, South Asia, and Sub-Saharan Africa.

### Statistical analysis

Categorical data are presented as absolute numbers and percentages. Comparison of descriptive data between groups was performed using $\chi$^2^ test and Fisher’s exact test as appropriate. A p value of < 0.05 was considered statistically significant. SPSS Version 24 (IBM SPSS, USA) was used for all analyses.

## Results

A total of 1150 participants from 109 countries completed the survey. Sixty-three percent of the participants were from high-income economies, 37% were from lower income economies (including low income, lower middle-income, and higher middle-income). Thirty-nine percent were cardiologists, 23% were sonographers, and 19% internal medicine specialists. Twenty-eight percent worked in university hospitals and 48% in non-tertiary hospital settings. Nineteen percent of the participants described themselves as beginners in echocardiography, 45% as moderately advanced, 29% as advanced, and 7% as experts. Eleven percent of the participants worked in the United States of America, 7% in Germany, 5% in Austria, 5% in India, and 5% in the United Kingdom. Full descriptive statistics are shown in Table 1.

**Table 1:** Baseline characteristics of the study population (n = 1150)

Baseline characteristics	All
Number of participants	1150
Age (years)	
< 30, n (%)	119 (10)
30–39, n (%)	418 (36)
40–49, n (%)	298 (26)
50–59, n (%)	200 (17)
60–69, n (%)	105 (9)
> 69, n (%)	10 (1)
Profession	
Sonographer, n (%)	264 (23)
MD, internal medicine, n (%)	214 (19)
MD, cardiologist, n (%)	447 (39)
MD, anesthesiologist, n (%)	53 (5)
MD, emergency n (%)	27 (2)
MD, intensive care, n (%)	51 (4)
MD, other doctor, n (%)	60 (5)
Other, n (%)	34 (3)
Work setting	
University hospital, n (%)	322 (28)
Hospital, n (%)	549 (48)
Private practice, n (%)	242 (21)
Other, n (%)	37 (3)
Level of expertise	
Beginner, n (%)	224 (19)
Moderately advanced, n (%)	519 (45)
Advanced, n (%)	329 (29)
Expert, n (%)	78 (7)
Number of echos performed	
< 100, n (%)	164 (14)
100–500, n (%)	232 (20)
501–1000, n (%)	178 (15)
1001–5000, n (%)	258 (22)
> 5000, n (%)	318 (28)
Country	
USA, n (%)	127 (11)
Germany, n (%)	85 (7)
Austria, n (%)	61 (5)
India, n (%)	56 (5)
United Kingdom, n (%)	52 (5)
Italy, n (%)	50 (4)
Poland, n (%)	37 (4)
Australia, n (%)	35 (4)
Sweden, n (%)	34 (3)
Greece, n (%)	33 (3)
Other countries, n (%)	580 (50)

*MD* medical doctor

### Individual diagnostic repertoire

Seventy-nine percent of the participants (n = 906) stated that they used eyeballing to assess right ventricular function. TAPSE was used by 82% of the participants, S′ by 48%, FAC by 26%, and RV-GLS by 11%. RIMP, 3D echocardiography, and dp/dt was used by 7, 4, and 6% respectively. Twenty percent of the participants used one method (including eyeballing) to assess right ventricular function, 29% used two methods, and 51% three or more methods.

### Methods regularly used in daily clinical routine

**Fig. 1:**
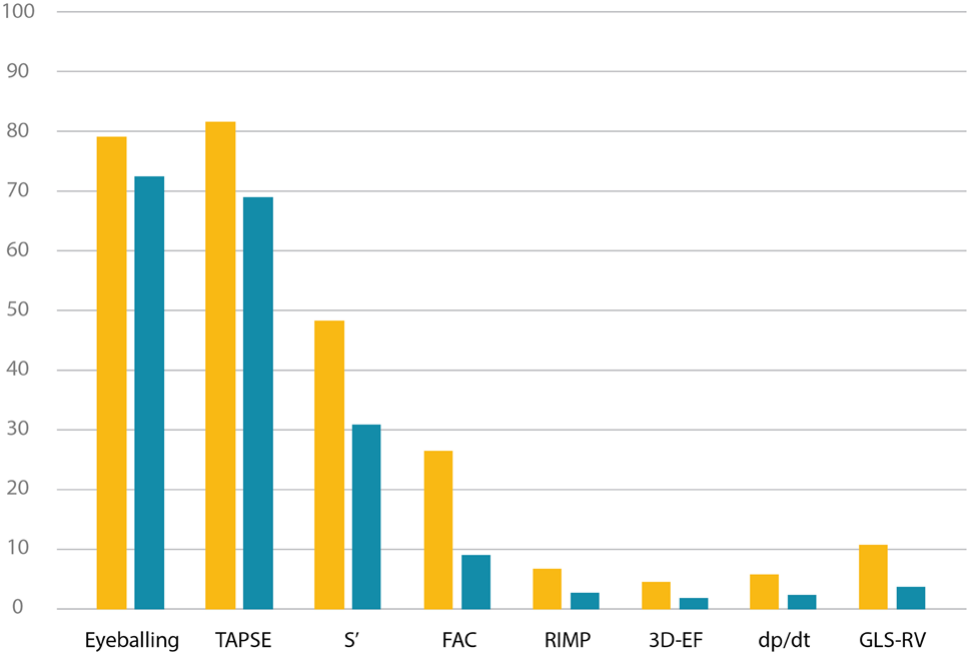
Methods used for right ventricular functional assessment: overall (yellow bar) and daily clinical routine (blue bar). *TAPSE* tricuspid annular plane systolic excursion, *S*′ tissue Doppler velocity of the basal free lateral wall of the right ventricle, *FAC* fractional area change, *RIMP* right ventricular myocardial performance index, *3D-EF* 3D ejection fraction, *Dp*/*dt* the rate of pressure rise in the RV, *GLS-RV* global longitudinal strain of the free wall of the right ventricle, *Y-axis* percent of the participants

Only eyeballing (72%), TAPSE (69%), and S′ (31%) were mentioned as frequently used methods. Thirty-nine percent of the participants applied only one method (including eyeballing) to assess right ventricular function in daily clinical practice, 35% used two methods, and 26% more than two methods. Eyeballing was applied as a single parameter by 23% of the participants (Table 2; Fig. 1).

**Table 2:** Parameters used in daily clinical routine for evaluation of right ventricular function

Methods applied in daily clinical routine	Total	University hospital	Others	p value
Eyeballing, n (%)	832 (72)	231 (72)	601 (73)	0.774
TAPSE, n (%)	798 (69)	241 (75)	557 (67)	0.012
S′, n (%)	353 (31)	108 (34)	245 (3)	0.192
FAC, n (%)	104 (9)	35 (11)	69 (8)	0.178
RIMP, n (%)	20 (2)	7 (2)	13 (2)	0.482
3D-EF, n (%)	13 (1)	5 (2)	8 (1)	0.398
dp/dt, n (%)	26 (2)	6 (2)	20 (2)	0.572
GLS-RV, n (%)	29 (3)	12 (4)	17 (2)	0.104

Methods applied in daily clinical routine	All	PH-center	Non-PH-center	p value
Eyeballing, n (%)	832 (72)	503 (73)	329 (72)	0.679
TAPSE, n (%)	798 (69)	488 (71)	310 (68)	0.266
S′, n (%)	353 (31)	245 (35)	108 (24)	< 0.001
FAC, n (%)	104 (9)	79 (11)	25 (5)	0.001
RIMP, n (%)	20 (2)	18 (3)	2 (0)	0.006
3D-EF, n (%)	13 (1)	10 (2)	3 (1)	0.213
dp/dt, n (%)	26 (2)	17 (2)	9 (2)	0.577
GLS-RV, n (%)	29 (3)	20 (3)	9 (2)	0.323

Comparison between different hospital settings

*TAPSE* tricuspid annular plane systolic excursion, *S*′ tissue Doppler velocity of the basal free lateral wall of the right ventricle, *FAC* fractional area change, *RIMP* right ventricular myocardial performance index, *3D-EF* 3D ejection fraction, *Dp*/*dt* the rate of pressure rise in the RV, *GLS-RV* global longitudinal strain of the free wall of the right ventricle

### University versus non-university setting

Significant differences were registered between those working in a university setting and those in non-university settings. TAPSE (p = 0.001), S′ (p = 0.01), FAC (p = 0.004), 3D-EF (p < 0.001), and RV-GLS (p < 0.001) were significantly more often mentioned as part of the individual diagnostic repertoire in the university setting. Those practicing at universities also used significantly (p < 0.001) more methods to assess RVF. However, the analysis of the respondents’ answers concerning daily clinical routine revealed no significant differences between the two settings (see Table 2).

### Experience with pulmonary hypertension patients

Sixty percent of the respondents stated that they regularly examined patients with severe pulmonary hypertension (PH). A significant (p < 0.001) difference was registered between the PH-center and the non-PH-center group with regard to the number of methods used in clinical routine. At PH centers, 31% of the respondents reported the use of three or more parameters (including eyeballing) to assess right ventricular function in clinical routine, as opposed to 16% at non-PH-centers. All imaging parameters were used more frequently at PH centers; the difference was statistically significant for S′ (p < 0.001), FAC (p = 0.001), and RIMP (p = 0.006). Full data about imaging at PH centers are summarized in Table 2.

### Cardiologists versus non-cardiologists

Eyeballing was used significantly more frequently by non-cardiologists, while TAPSE was used significantly more often by cardiologists. The differences in all other parameters did not reach statistical significance (data not shown).

### Differences between high income and low income economies

Results were compared between high income economies on the one hand and low income, lower middle-income, and upper middle-income on the other hand. There were significant differences between the two groups. While eyeballing, TAPSE, S′, and FAC are the four most commonly applied parameters in both groups, all of the methods were applied significantly more often in the high-income economies as opposed to the lower income economies (data not shown).

### Differences between regions of the world

**Fig. 2:**
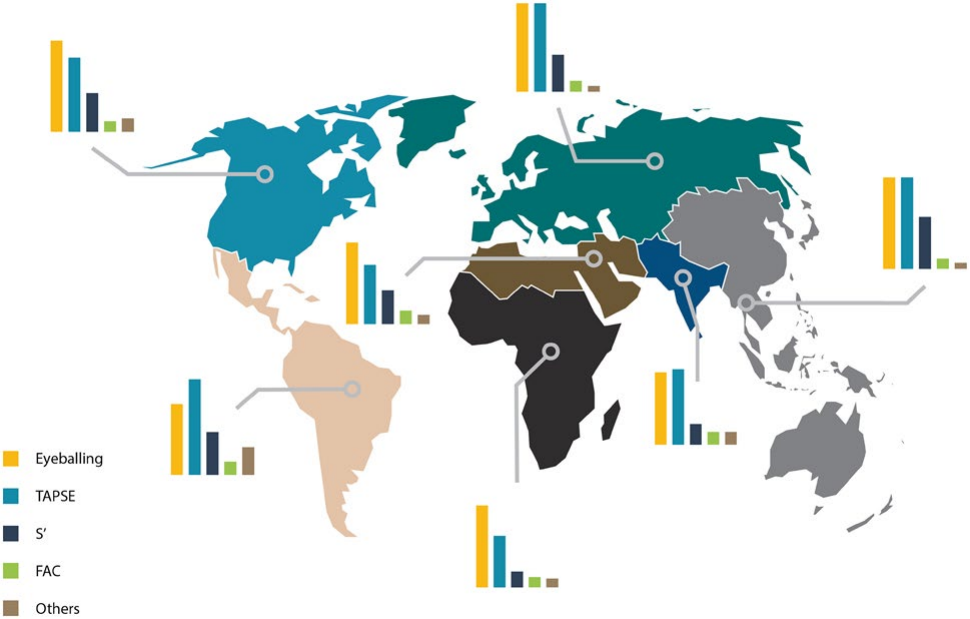
Regional differences in right ventricular functional assessment. *TAPSE* tricuspid annular plane systolic excursion, *S*′ tissue Doppler velocity of the basal free lateral wall of the right ventricle, *FAC* fractional area change

Eyeballing (72%) and TAPSE (69%) are the most commonly applied parameters in all regions of the world. TAPSE is used less frequently in the Middle East and North Africa (49%) and in Sub-Saharan Africa (44%), S′ is used less frequently in South Asia (17%) and in Sub-Saharan Africa (13%). Furthermore, there were differences in the number of parameters applied for RV function gradation. Eyeballing as a single parameter was used in less than 20% of the participants from East Asia and Pacific, Europe and Central Asia, and Latin America and the Caribbean, and by 30% of those from North America, by 37% by those from the Middle East and North Africa, by 28% of the participants from South Asia, and by 48% of those from Sub-Saharan Africa (Fig. 2).

## Discussion

To our knowledge this is the first evaluation of clinical practice concerning the assessment of right ventricular function by echocardiography in a large group of health care professionals (medical doctors and sonographers) on a global scale. Eyeballing (79%) and TAPSE (82%) were the most commonly used parameters, followed by the tissue Doppler parameter S′, which was applied by 31% of respondents. All other parameters were used infrequently. Notably, the novel parameters such as RV-GLS and 3D echocardiography have not yet been established in clinical routine—neither in the academic setting nor in non-tertiary hospitals. Guidelines recommend that, in addition to eyeballing, at least one other parameter should be used to assess right ventricular function. Eyeballing alone is not sufficient for the quantification of right ventricular function [1, 2]. However, only 77% of our respondents adhered to this recommendation.

This gap between current scientific knowledge and actual real-world echocardiography deserves attention and should be addressed by the echocardiographic community.

### Tricuspid annular plane systolic excursion and tissue Doppler imaging

TAPSE and S′, as measures of longitudinal motion of the basal lateral free wall of the RV, have several limitations. They only reflect the motion of the basal segments of the RV and may lead to a significant over- or underestimation of right ventricular function in the presence of regional differences in contraction. Displacement of the apex, resulting in passive motion of basal right ventricular segments, may result in the overestimation of RVF using TAPSE. TAPSE does not reflect true right ventricular function in many clinical conditions, including patients who have undergone cardiac surgery, those with Tetralogy of Fallot, and those with tricuspid regurgitation [22–25]. Apart from eyeballing, TAPSE (69%) and S′ (31%) are the most commonly used parameters for RVF gradation. This may be due to the fact, that TAPSE and S′ are simple parameters, can be applied rapidly, and do not require sophisticated technology.

### Fractional area change

FAC is a quantitative parameter that reflects volume change during contraction. Nine percent of our respondents used this parameter in clinical routine. The meager use of this procedure is surprising in view of the fact that FAC can be measured with every echo machine; no Doppler recordings or additional software is needed. Moreover, the clinical value of the parameter has been confirmed in several studies [11–14].

Eleven percent of those who worked at PH centers used FAC in clinical routine, as opposed to 5% at non-PH centers (p = 0.001). The significantly more frequent use of FAC at PH centers indicates the valuable additional information provided by this method, especially in patients with severely dilated right ventricles and reduced FAC, but preserved longitudinal contraction. The rare use of the method at non-PH centers may be explained by the additional time taken to perform the procedure, and uncertainty about correct tracing of the right ventricular endocardial border.

### Myocardial performance index and the rate of pressure rise in the right ventricle

RIMP and dP/dt reliably predict reduced right ventricular function. Among our echocardiographers, both methods were used by a mere 2% in clinical practice. Dp/dt and RIMP require the recording of special Doppler signals which might not be a part of the standard imaging protocol at many echocardiography laboratories. Acquisition is not as straightforward as the simple measurement of distance (TAPSE), area (FAC), or peak velocity (S′). The infrequent use may be related to difficulties in understanding physiologic and pathologic principles as well as the additional time taken for the assessment.

### New technologies

As expected, new and advanced parameters of RV quantification are used more frequently in the university setting than in the non-university setting. However, the difference did not persist in daily clinical routine.

Longitudinal strain of the free lateral wall of the RV is a promising new technology to asses RVF. It measures the deformation of the myocardium and permits regional as well as global assessment of the free lateral wall of the right ventricle. RV longitudinal strain is occasionally used by 11%, and regularly used by 3% in clinical routine. Strain analysis requires dedicated software, good image quality, ECG tracing, and additional time for analysis. The method is easy to use but expensive. Our data showed that although strain analysis has been available for several years, it is rarely used in daily clinical practice.

3D volume assessment yields end-diastolic and end-systolic volumes as well as ejection fraction and stroke volume. It is not affected by regional variations in RV contractility or passive right heart motion. 3D echocardiography was occasionally used by 4% of the respondents and regularly used by 1% in clinical routine. 3D echocardiography requires dedicated software and hardware, which may not be available at many centers. Advanced and expensive imaging systems are available at university hospitals and usually do not exist in smaller hospitals or medical offices. Thus, it was no surprise to note that 3D echocardiography is available more frequently in university hospitals than in other settings (8 vs. 3%, p < 0.001). Time and experience also play a role. A multi-beat 3D dataset of the right ventricle including offline analysis requires significantly more time than TAPSE and S′ and—in addition—the skill to acquire loops with very good image quality. It is used significantly less often by beginners than by advanced echocardiographers.

RV-GLS and volume assessment of the RV by 3D echocardiography are promising new tools for the assessment of right ventricular function. The present investigation showed that significant effort will be needed in the future to implement these methods in daily routine. Echocardiographers will have to be trained in, and convinced of, the use of these parameters. Recent promising developments in 3D technology have accelerated and improved the acquisition and interpretation of RV-3D datasets by introducing a single-beat method for RV volume analysis [26]. The industry should now focus on further simplification of the new methods and the development of affordable algorithms.

### Differences between regions of the world and different levels of economic strength

Eyeballing and TAPSE are the most commonly applied parameters in all regions of the world. TAPSE is used less frequently in the Middle East and North Africa and in Sub-Saharan Africa, S′ is used less frequently in South Asia and in Sub-Saharan Africa.

There were significant differences between high-income and lower income economies. While eyeballing, TAPSE, S′, and FAC are the four most commonly applied parameters in both groups, all of the methods were applied significantly more often in the high-income economies as opposed to the low-income economies. As TAPSE and FAC are parameters solely based on 2D measurements which can be performed by every echo machine, higher costs for more sophisticated imaging systems do not explain the observed differences. The differences can be explained through less reachable training in echocardiography, different health care systems with less capacities for the diagnosis and treatment of complex diseases and therefore more focused examinations.

### Limitations

Data were collected anonymously through an online survey. Therefore, accuracy of the individual responses could not be verified; we had to rely on the honesty of the participants. However, it may be assumed that the results overestimate true clinical practice and not vice versa. The anonymous nature of the survey permitted a large number of persons to participate in the study. The study participants were recruited from the users of an online teaching platform, which may have signified a selection bias in favor of echocardiographers interested in continuing medical education and new technologies. Again, it may be assumed that the results overestimate the application of parameters of right ventricular function.

Due to the mode of recruitment this study analyzes a heterogenic sample. Participants were contacted through the network of an online teaching website, including social media platforms. Therefore, the rate of participation in relation to those contacted cannot be determined. Thus, the analyzed group is not a representative sample but rather a large real-life group of echocardiographers. Considering the homogenous results showing a similar distribution of applied methods in all countries, professional groups, and levels of expertise, it can be assumed that the presented data nevertheless represents the real world daily clinical practice.

More information on the reasons for the meager use of modern parameters would have been of great interest. This survey focused on the assessment of the current state of clinical practice, further questions regarding reasons for certain answers were not asked. The nature of the short questionnaire allowed for a large number of participants.

## Conclusions

Eyeballing and TAPSE may lead to misinterpretations of right ventricular function. However, the two parameters are applied most frequently for the quantification of right ventricular function. New methods such as 3D echocardiography and RV-GLS are employed by some physicians, mainly in the university hospital setting. Although these advanced parameters were shown to be highly accurate, they are rarely used in daily clinical routine.

Our investigation revealed that the assessment of right ventricular function by health care professionals does not conform to current guidelines. This is especially true of the non-academic setting. It therefore remains the task of the echocardiographic community to continue teaching about the importance of thorough assessment of right ventricular function.
